# Genome Sequences of Two Gordonia rubripertincta Cluster DJ Bacteriophages, Pherobrine and Burley

**DOI:** 10.1128/mra.01024-22

**Published:** 2022-11-08

**Authors:** Aaron J. Adams, Michael Bell, Marie J. Bennett, Umar N. Chaudhry, Jennifer N. Daniels, James E. Herman, Sophia A. Oh, Fedora Parra, Cynthia R. Reagan, Borna Zareiesfandabadi, Marie P. Fogarty

**Affiliations:** a Science Department, Durham Technical Community College, Durham, North Carolina, USA; DOE Joint Genome Institute

## Abstract

Pherobrine and Burley are siphoviruses infecting Gordonia rubripertincta. Pherobrine has a 60,305-bp genome with 89 predicted protein-coding genes, and Burley has a 60,111-bp genome with 90 predicted protein-coding genes. Both phages are assigned to cluster DJ, where they share 78% gene content similarity with each other.

## ANNOUNCEMENT

The isolation and genomic characterization of novel bacteriophages can support the development of therapeutics to address the global increase in antibiotic-resistant bacterial infections (1). Here, we report the isolation and genome sequence of two lytic bacteriophages, Pherobrine and Burley.

**FIG 1:**
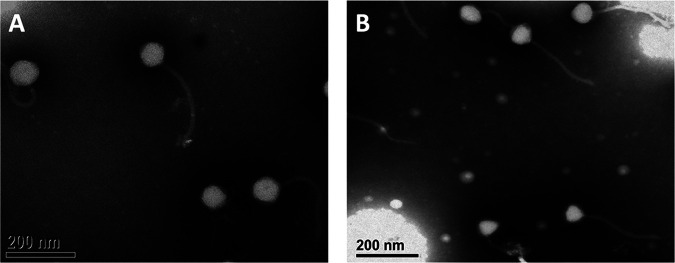
Transmission electron microscopy images of Pherobrine (A) and Burley (B).

Pherobrine and Burley were extracted from soil samples collected in August 2021 in Durham (36.118889°N, 78.864722°W) and Cedar Grove (36.2209°N, 79.16892°W), North Carolina, respectively, using Gordonia rubripertincta NRRL B-16540 and standard isolation procedures (2). Briefly, the soil samples were washed with peptone-yeast extract-calcium (PYCa) liquid medium, the wash was filtered through 0.22-μm filters, and the filtrate was inoculated with G. rubripertincta and incubated with shaking at 30°C for 3 to 5 days. An aliquot of the culture was filtered and diluted, and dilutions were plated in top agar containing G. rubripertincta and incubated at 30°C. Both phages, which produced clear plaques with an ~1-mm-wide diameter after 3 days at 30°C, were purified through three rounds of plating. Lysates were negatively stained using 2% phosphotungstic acid (PTA) and imaged by transmission electron microscopy (TEM) to reveal siphovirus morphologies. The capsid diameter was 72 to 80 nm (*n* = 5 for each phage), while the average tail length was 240 to 264 nm (*n* = 5 for each phage) (Fig. 1).

DNA was extracted from Pherobrine and Burley using phenol-chloroform-isoamyl alcohol extraction (Sigma-Aldrich; catalog no. P2069), prepared for sequencing using the NEBNext Ultra II library kit, and sequenced using the Illumina MiSeq (v3 reagents) platform to yield 150-bp single-end reads that provided 1,543-fold genome coverage of Pherobrine and 360-fold genome coverage of Burley. The reads were assembled using Newbler v2.9 and checked for accuracy, coverage, and genomic termini using Consed v29 (3–5). The sequencing results and genome characteristics are listed in Table 1. Based on gene content similarity (GCS) of at least 35% to phages in the Actinobacteriophage database, phagesDB (6), both Pherobrine and Burley, which share 78% GCS, were assigned to phage cluster DJ.

**TABLE 1:** Genome assembly results for Pherobrine and Burley

Phage name	No. of reads	Avg fold coverage	Genome size (bp)	Genome ends	% GC content	Cluster assignment
Pherobrine	622,045	1,543	60,305	3′ Single-stranded overhang (5′-CGCCGCCCT-3′)	51.8	DJ
Burley	1,016,953	360	60,111	3′ Single-stranded overhang (5′-CGCCGCTCT-3′)	51.6	DJ

The genomes of Pherobrine and Burley were auto-annotated using Glimmer v3.02 (7) and GeneMark v2.5p (8). Manual inspection and annotation refinement were carried out using DNA Master v5.23.6 (9), PECAAN (https://discover.kbrinsgd.org/), Phamerator (10), Starterator v1.2 (9), NCBI BLASTp (11), and HHpred v3.2 (PDB, CDD, pFamA, UniProt) (12). TMHMM v2.0 (13) and SOSUI v1.11 (14) were used for transmembrane domain prediction, and ARAGORN v1.2.38 (15) and tRNAscan-SE v2.0 (16) were used for tRNA prediction. Default parameters were used for all software. Pherobrine and Burley contain 89 and 90 genes, respectively, all of which are transcribed rightward. Genes encoding structural, assembly, and lysis functions are encoded on the left half of the genome, whereas DNA metabolism functions are encoded on the right arm of the genome. At least 12 membrane proteins were predicted in each phage, while no tRNA, immunity repressor, or integrase genes could be identified. Notably, and characteristic of cluster DJ phages, a region spanning ~7 kbp downstream of tape measure gene contains multiple sequence repeats that occur intergenically and upstream of ATG gene start sites, suggesting an involvement in gene regulation.

### Data availability.

Pherobrine is available at GenBank with accession no. ON970572 and Sequence Read Archive (SRA) with accession no. SRX14483240, and Burley is available at GenBank with accession no. ON526971 and SRA with accession no. SRX15121737.
